# Video Q&A: molecular profiling of breast cancer

**DOI:** 10.1186/1741-7015-11-150

**Published:** 2013-06-18

**Authors:** Carlos Caldas

**Affiliations:** 1 Cancer Research, UK Cambridge Institute, New Orleans, LA, 70124, USA

## Abstract

In this video Q&A, we talk to Professor Carlos Caldas about the identification of breast cancer subtypes through molecular profiling, and the clinical implications for diagnosis and treatment.

## Carlos Caldas talks on molecular profiling of breast cancer

## Introduction

Carlos Caldas is a Professor of Cancer Medicine at the University of Cambridge in the UK, and currently heads the Breast Cancer Functional Genomics Laboratory at the Cancer Research UK Cambridge Institute. He is an Honorary Consultant Medical Oncologist at Addenbrooke’s Hospital and his research interests are in breast cancer functional genomics, with the aim to identify novel predictive markers and therapeutic targets. Recently he led a landmark study classifying breast cancer into 10 different subtypes, which could revolutionize the way breast cancer is treated.

## Edited transcript

### 1. How is breast cancer currently managed?

The first thing to do is to diagnose breast cancer. In countries like the UK with screening programs, there is high awareness of breast cancer and it is often diagnosed at an early stage. After diagnosis with a biopsy, the cancer is usually removed by surgical resection. We try to use breast-conserving surgery rather than mastectomy to try to preserve the breast. Most patients undergoing breast-conserving surgery also have radiotherapy to prevent recurrence. It is then necessary to decide which adjuvant systemic treatment to give; women are risk stratified into different groups based on whether they are going to benefit from adjuvant therapy, particularly chemotherapy. Two other forms of adjuvant therapy are used; hormone therapy for women who have estrogen receptor positive breast cancer (at least 75% of all breast cancer), and the monoclonal antibody trastuzumab for HER2 positive breast cancer (up to 15% of all breast cancer).

### 2. What are the problems with current treatments for breast cancer?

The problems are that any systemic treatment, whether that is hormone therapy, chemotherapy or the more targeted monoclonal antibody therapy, carries costs and has the potential for toxicity and harming the patient. Therefore, we try to make decisions that are rational and maximize benefit for women that have higher risk. In the case of hormone therapy and trastuzumab, we have very good predictive tests that can help to assess benefit. For example, we know that women with estrogen receptor negative breast cancer do not benefit from hormone therapy, and those with HER2 negative breast cancer do not benefit from trastuzumab treatment. We therefore have two tests that are routinely applied in the clinic to make decisions for these two therapies. It is more difficult to decide whether to prescribe adjuvant chemotherapy because we don’t have any specific tests to tell us whether it will be effective, and we currently make decisions based on risk profiling using diagnostic tests applied in the clinic. The promise of novel tests comes from molecular characterization of tumors, which will make these decisions more rational, quantitative and precise.

### 3. What have been the major breakthroughs in breast cancer research over the last couple of years?

Until recently we did a couple of tests to help make decisions on breast cancer management. Major determinants of prognosis assessed by pathologists are tumor grade, tumor size and lymph node involvement. Two immunohistochemical tests are then done, one for the estrogen receptor and one for HER2, to help predict benefits from the therapies discussed earlier. These tests are done in routine clinical practice, and it is possible to divide breast cancer into four groups based on immunohistochemistry:

1. Estrogen receptor positive and HER2 negative (around 70% of breast cancers)

2. Estrogen receptor and HER2 positive (around 7.5% of breast cancers)

3. Estrogen receptor negative and HER2 positive (around 7.5% of breast cancers)

4. Negative for both estrogen receptor and HER2 (the remaining 15% of breast cancers)

What we have now shown with our work is that breast cancer is at least 10 different diseases, and so we can be much more precise in risk stratifying, as well as developing novel therapies and estimating whether a given treatment is going to be of benefit for a particular patient.

### 4. How will the findings of your study on breast cancer subtypes be extended?

Our findings published in Nature in 2012 clearly show that breast cancer is 10 different diseases. The study was very robust because the subtypes were identified in 1000 breast cancers and then further confirmed in another 1000. Since then, The Cancer Genome Atlas (TCGA) group in America confirmed that they could also identify these 10 subtypes. Therefore, we now have robust evidence that these 10 subtypes have different outcomes, and it is likely that they would benefit from different forms of treatment. We now need to translate this into clinical application. My group and others worldwide are trying to come up with a test that assigns women to one of the 10 classes upon breast cancer diagnosis. This work is going to occur over the next 2 or 3 years, and we hope to offer it as a diagnostic test in the future.

### 5. How will the findings be applied in the clinic?

I think women will be assigned to these 10 different classes because they all have a very different prognosis. There are classes where the survival rate is as good as over 90%. There are other classes where unfortunately the survival with current therapies is poor. In groups with very good survival we can start asking whether treatments with toxic side effects can be withheld because women with very good prognosis might not benefit from them. On the other hand, women with very poor prognosis could be prioritized for trials with new drugs. This is one of the more obvious clinical applications of this information.

### 6. Have you encountered any ethical issues in your research into breast cancer genetics?

I think that whenever we do studies involving genetics and patients’ clinical outcome, we need to be very guarded and protect the autonomy of the individuals participating in the studies. We can only recruit participants if they give appropriate consent. After having protected each patient’s right to autonomy, we also need to respect their privacy and their right to not having their information displayed in public. We have to be very careful that when we report these studies, personal identifiers are completely removed from the data that is publicly available. Proper consenting means explaining to the participants that we are going to be obtaining genetic information but also reassuring them that we will do the utmost to protect their privacy and anonymize their data, so that people feel confident continuing to participate in these studies.

### 7. Where do you think breast cancer stands in terms of patient-tailored therapy in comparison with other types of cancer?

I think that it depends on how you look at it. Being a very common malignancy, we already use predictive tests routinely in breast cancer. As I explained earlier, we do the two tests for estrogen receptor and HER2 in the NHS, which are extremely important in determining treatments with hormone therapy and trastuzumab, respectively. Where we are still falling short is in having tests that do likewise for chemotherapy. At the moment we do not have tests that can tell us whether or not women will benefit from chemotherapy, and we clearly need to develop those tests. We also hope to develop tests to tell us if all current treatments are going to be of real benefit, and to show which patients should be prioritized for studies of novel compounds. I think that this is where breast cancer stands at the moment; there is lots of advance on one hand but also lots of work to do on the other.

### 8. Where can I find out more?

See reference list [1-9]

## Pre-publication history

The pre-publication history for this paper can be accessed here:

http://www.biomedcentral.com/1741-7015/11/150/prepub

## Supplementary Material

Additional file 1 Carlos Caldas talks on molecular profiling of breast cancer. Click here for file
